# Restricted Expression of Epstein-Barr Virus Latent Genes in Murine B Cells Derived from Embryonic Stem Cells

**DOI:** 10.1371/journal.pone.0001996

**Published:** 2008-04-16

**Authors:** Magdalena Zychlinska, Heidrun Herrmann, Ursula Zimber-Strobl, Wolfgang Hammerschmidt

**Affiliations:** 1 Department of Gene Vectors, Helmholtz Center Munich, German Research Center for Environment and Health, Munich, Germany; 2 Institute for Clinical Molecular Biology and Tumor Genetics, Helmholtz Center Munich, German Research Center for Environment and Health, Munich, Germany; The Rockefeller University, United States of America

## Abstract

**Background:**

Several human malignancies are associated with Epstein-Barr virus (EBV) and more than 95% of the adult human population carries this virus lifelong. EBV efficiently infects human B cells and persists in this cellular compartment latently. EBV-infected B cells become activated and growth transformed, express a characteristic set of viral latent genes, and acquire the status of proliferating lymphoblastoid cell lines *in vitro*. Because EBV infects only primate cells, it has not been possible to establish a model of infection in immunocompetent rodents. Such a model would be most desirable in order to study EBV's pathogenesis and latency in a suitable and amenable host.

**Methodology/Principal Findings:**

We stably introduced recombinant EBV genomes into mouse embryonic stem cells and induced their differentiation to B cells *in vitro* to develop the desired model. *In vitro* differentiated murine B cells maintained the EBV genomes but expression of viral genes was restricted to the latent membrane proteins (LMPs). In contrast to human B cells, EBV's nuclear antigens (EBNAs) were not expressed detectably and growth transformed murine B cells did not arise *in vitro*. Aberrant splicing and premature termination of *EBNA* mRNAs most likely prevented the expression of *EBNA* genes required for B-cell transformation.

**Conclusions/Significance:**

Our findings indicate that fundamental differences in gene regulation between mouse and man might block the route towards a tractable murine model for EBV.

## Introduction

Epstein-Barr virus (EBV) is a human herpesvirus, which has been categorized as the first known human tumor virus [1]. This virus is widespread and persists in all infected individuals as a lifelong, usually asymptomatic and latent infection in the B-cell compartment. Acute infection with EBV can cause infectious mononucleosis, and its latent state can evolve to yield several B-cell lymphomas, nasopharyngeal carcinoma and gastric cancer, and other more sporadic malignancies [2], [3]. Although EBV has been studied extensively at the molecular level *in vitro*, conditions of virus latency, virus-associated diseases and their pathogenesis are difficult to study in the human population due to individual variations in genetics, environment and behaviour.

EBV is highly species-specific in that it infects human and certain primate cells, only. Much effort has gone into establishing a suitable animal model for EBV but even the most advanced models, i.e. humanized mice show considerable limits [4]. Experimental infections of mice with an EBV-related murine herpesvirus termed MHV68 share certain features of EBV's pathogenesis and latency [5] but MHV68 does not encode homologues of EBV latency-associated or transforming genes. These EBV genes include the latent membrane proteins (*LMP)1* and *LMP2a* as well as the EBV nuclear antigens (*EBNA)2*, and *EBNA1*, among others. All of these latent gene products have intrinsic activating characteristics [2], [6]–[10] and thereby contribute to growth transformation of human B cells, an accepted *in vitro* model, which partially reflects EBV's contributions to viral oncogenesis.

A mouse model that embodies multiple features of EBV's infection would be most desirable to understand EBV's contribution to viral latency and tumor development. In addition, combining mouse genetics and viral mutants would open the opportunity to unravel the relationship of the virus with its host at the genetic and molecular level. EBV-susceptible transgenic mice or primary murine cells, which carry a priori defined genetic mutations in cellular genes presumably involved in EBV's pathogenesis, would be valuable tools and greatly advance our understanding of the functions of cellular and viral gene products and their critical interplay.

There are multiple viral and host restrictions, which uniquely direct EBV's susceptibility to humans including the human surface receptor CD21 and HLA class II molecules, which are crucially involved in virus adsorption and entry [11], [12]. Recently, it has been shown that murine lymphoma cell lines stably transfected with both human genes became permissive to EBV and maintained the viral genome stably in an extrachromosomal and latent form [13]. Clearly, the infection rates were low and all efforts to infect primary B cells derived from transgenic mice, which express these two human surface molecules, have failed so far [14] (R. Longnecker, personal communication). We, therefore, followed a different route and introduced recombinant EBV genomes into murine embryonic stem cells (mESCs) and induced their differentiation to murine B cells *in vitro* in order to study EBV and its functions in non-transformed mouse B cells.

## Results

### Establishment of mESC lines expressing EBNA1

**Figure 1:**
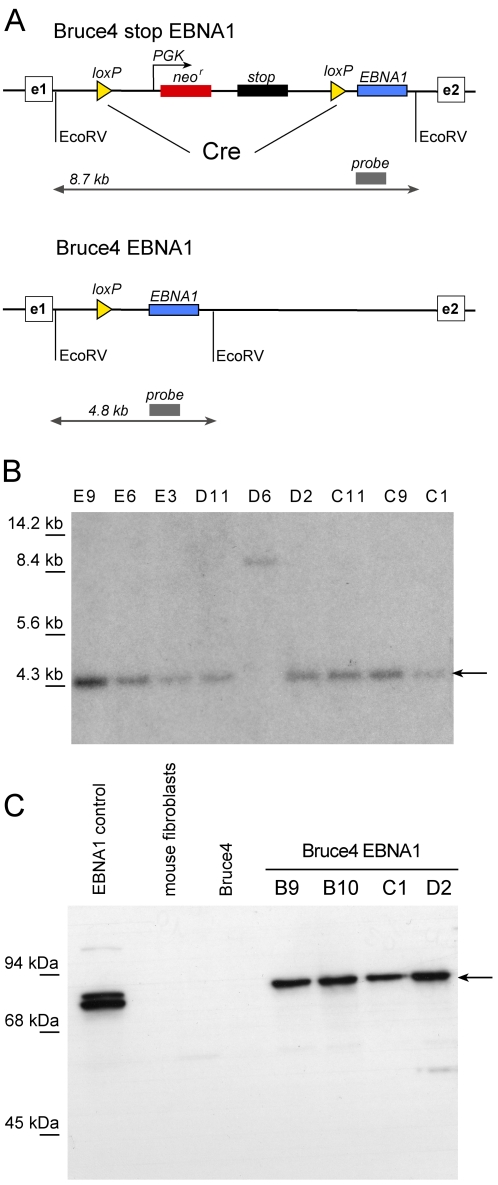
Targeting of *EBNA1* into the *rosa26* locus. (A) Molecular cloning strategy. A loxP flanked transcriptional stop cassette (*stop*) and the selectable maker gene for G418 resistance (*neo^r^*) precede the *EBNA1* gene in the rosa26 intron bracketed by exon 1 (e1) and exon 2 (e2) (top). After Cre-mediated deletion *EBNA1* is transcribed from the ubiquitously expressed *rosa26* locus in Bruce4 EBNA1 cells (bottom). (B) Southern blot analysis of several Bruce4 EBNA1 cell lines after Cre-mediated deletion of the *stop* cassette. The radioactive probe, depicted in (A), distinguishes between the initial targeted situation (8.7 kb) and deletion of the stop cassette (4.8 kb). (C) Expression of EBNA1 in different Bruce4 EBNA1 cell lines as confirmed by immunodetection (arrowhead). EBNA1-positive 293 cells serve as positive control, negative controls are mouse embryonic fibroblasts and parental mESCs (Bruce4).

In preliminary experiments (discussed below), we noticed that mESCs did not survive selection when transfected with recombinant EBV genomes encoding the G418 resistance gene *neo*. Because nuclear retention and DNA replication of extrachromosomal EBV genomes depend on expression of the viral *EBNA1* gene we considered that it might be expressed at insufficient levels in mESCs. Therefore, we stably introduced *EBNA1* into the mouse *rosa26* locus. This locus is known to assure stable expression of inserted genes in all cell types [15]. Our genetic strategy is outlined in Fig. 1A. *EBNA1* was integrated into the first intron of the *rosa26* gene in Bruce4 mESCs in two steps. First, the locus was targeted with the linearized p3032 plasmid fragment carrying *EBNA1* with a preceding transcriptional stop cassette encompassing the *neo* gene. The stop cassette was flanked by two *loxP* sites oriented in the same direction. After successful transfection and selection, 400 G418-resistant single cell clones were obtained and analyzed for correct homologous recombination by Southern blot hybridization. Several correct clones were identified. In the second step, Cre recombinase was transiently expressed in one of the mESC clones, which led to the deletion of the stop cassette in 17 out of 20 clones analyzed (Fig. 1A). Southern blot hybridizations confirmed this genetic manipulation and concomitant removal of the *neo* gene (Fig. 1B). Expression of EBNA1 was assessed by Western blots in nine clones, four of which are shown in Fig 1C.

### Introduction and maintenance of EBV genomes in EBNA1-expressing mESCs

**Figure 2:**
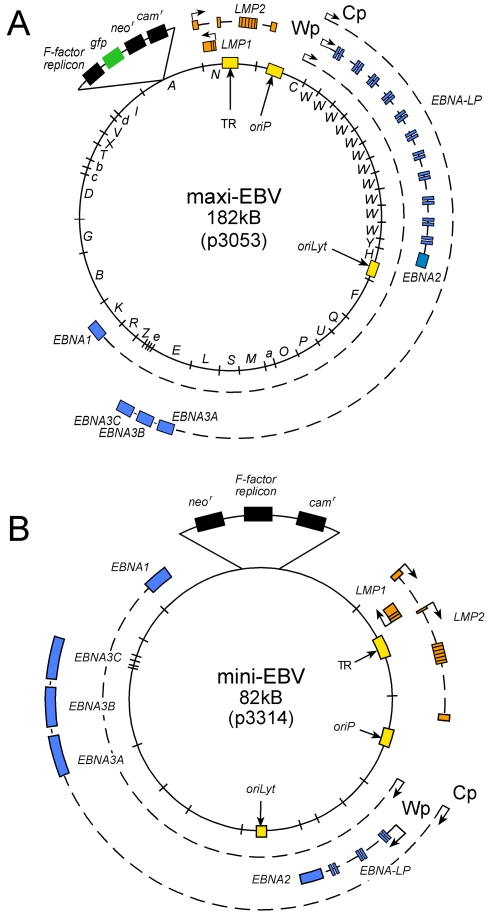
Recombinant EBV genomes. The maxi-EBV construct p3053 encompasses the prototypic EBV genome of the B95.8 strain [16], while the mini-EBV constructs p3314 lacks most lytic genes but encodes all latent viral genes [17]. Both plasmids can be selectively propagated in bacteria (F-factor replicon) under chloramphenicol selection (*cam^r^* in p3314, not marked in p3053) and in eukaryotic cells under G418 selection (*neo^r^*). p3053 also encodes *gfp*. Only latent viral genes are depicted, blue stands for *EBNA* genes and orange for *LMP* genes. Selected promoters are shown with arrowheads. ‘W’ stands for BamHI-W-repeats; *oriP* stands for the plasmid origin of replication, *oriLyt* is the active replication origin during virus synthesis. Terminal repeats (TR) are indispensable for encapsidation of virion DNA.

One EBNA1^+^ mESC clone, Bruce4 EBNA1 C1 (Fig. 1B, C), was electroporated with three different recombinant genomic EBV plasmids. Plasmid p3053 contains the complete EBV genome of the prototypic EBV strain B95.8, which was cloned onto a mini-F-plasmid in *E.coli*. Like its parental version p2089 [16], p3053 encodes *gfp* but in addition carries the G418 resistance gene *neo* located in the prokaryotic backbone (Fig 2A). This maxi-EBV plasmid is fully competent to give rise to progeny virus when propagated in appropriate cells or can directly mediate growth transformation of human B cells upon DNA transfection [16], [17]. p3298 is a variant of p3053 that is incapable of expressing EBV's latent genes other than *LMP2B* and *EBERs* and was used as a negative control (not shown). The mini-EBV plasmid p3314 is approximately half the size of a wild-type EBV genome, lacks the majority of EBV's lytic genes required for *de novo* virus synthesis (Fig. 2B) but carries all known latent EBV genes. This recombinant EBV genome is capable of transforming primary human B cells as efficiently as wild-type EBV [17], [18].

**Figure 3:**
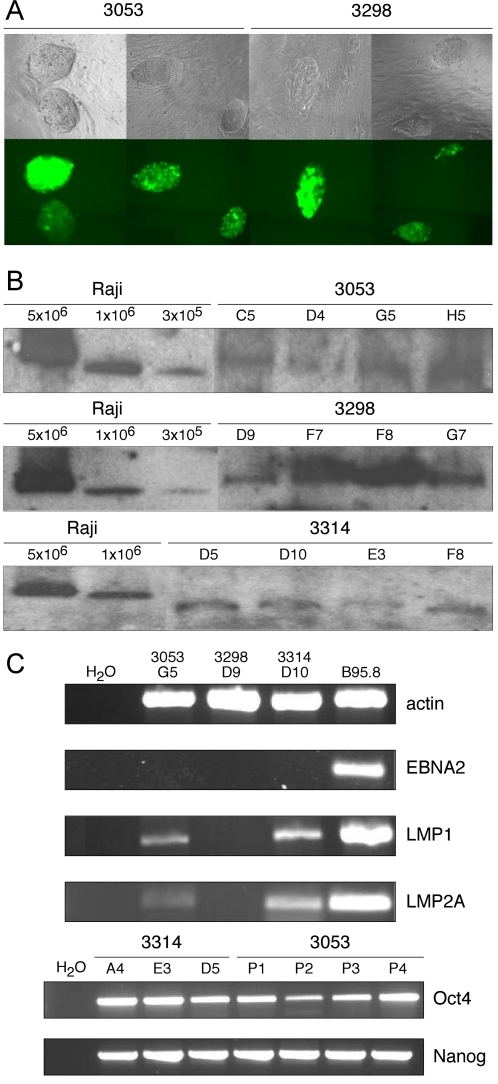
mini- and maxi-EBVs in Bruce4 EBNA1 mESCs. (A) GFP expression of the stably introduced maxi-EBV constructs p3053 and p3298 in Bruce4 EBNA1 cells. (B) Gardella gel hybridizations reveal the extrachromosomal status of the recombinant EBV genomes. Raji cells, which contain about 50 EBV genomes per cell served as a positive control. In each single lane, 5×10^6^ Bruce4 EBNA1 cells carrying different recombinant EBVs as indicated were loaded. (C) Detection of cellular transcripts (actin, Oct4, Nanog) and viral transcripts (EBNA2, LMP1, LMP2) by RT-PCR amplification. Selected Bruce4 EBNA1 clones stably transduced with the indicated recombinant EBV constructs are shown.

The recombinant maxi- and mini-EBV plasmids were separately introduced into the mESC clone Bruce4 EBNA1 C1 and selected with G418. Cells derived from several independent experiments were phenotypically and genotypically analyzed. GFP expression could be detected in mESCs electroporated with p3053 or p3298 (Fig. 3A) and the extrachromosomal status of the recombinant EBV genomes in all cell lines was confirmed by Gardella gel analysis (Fig. 3B). With this technique, intact large extrachromosomal EBV plasmids can be separated from genomic DNA and detected by Southern blot hybridization. Clearly, this technique does not exclude the possibility of integrated EBV DNA also present in the selected mESCs. In summary, the Bruce4 EBNA1 C1 mESC clone readily supported the maintenance of all different EBV plasmids, whereas the parental *EBNA1*-negative Bruce4 cells did not (data not shown).

### Selected viral and cellular genes expressed in EBV^+^ mESCs

We asked whether EBV promoters and cellular genes characteristic of embryonic stem cell genes were transcribed in mESCs cells stably carrying the recombinant EBV genomes. Using conventional and semi-quantitative real-time PCR we investigated the expression of three key viral genes: *EBNA2, LMP1*, and *LMP2A*, all involved in EBV-induced B-cell growth transformation, and two cellular genes, *Oct4* and *Nanog*, required for pluripotency and primarily expressed in undifferentiated ESCs [19]. Upon reverse transcription of total cellular RNA, cDNAs were amplified with specific primers designed to span at least one intron, in order to discriminate between amplification of cDNA or the genomic DNA template (Table S1). In all mESC lines carrying maxi-EBV p3053 or mini-EBV p3314 genomes, *Oct4, Nanog*, and both *LMP* genes were expressed but *EBNA2* transcripts were not detected (Fig. 3C; Table 1). The LMP1 gene product could not be detected directly in immunoblots indicating that its expression level was very low (data not shown). Alternatively, failure to detect LMP1 protein could be due to aberrant mRNA splicing of its transcript (see below). As expected, the control maxi-EBV p3298 did not show transcription of any of these latent viral genes (Fig. 3C and data not shown).

**Table 1:** Results of RT-PCR analysis of mESC lines stably transduced with two recombinant EBV genomes.

	EBV DNA	GAPDH	EBNA2	LMP1	LMP2A
Bruce4 EBNA1 3314 A4	+	+	–	+	+
Bruce4 EBNA1 3314 E3	+	+	–	+	+
Bruce4 EBNA1 3314 D5	+	+	–	+	+
Bruce4 EBNA1 3053 P1	+	+	–	+	+
Bruce4 EBNA1 3053 P2	+	+	–	+	+
Bruce4 EBNA1 3053 P3	+	+	–	+	+
Bruce4 EBNA1 3053 P4	+	+	–	+	+
B95.8	+	+	+	+	+

(+) indicates detection of cDNA of a given transcript, minus (–) its absence. B95.8 cells serve as positive control for EBV's latent genes. The gene encoding glyceraldehyde 3-phosphate dehydrogenase (GAPDH), is an ubiquitously expressed housekeeping gene, which serve as another positive control for correct cDNA synthesis and PCR amplification. EBV (+) indicates detection of genomic EBV DNA. Primers used are listed in Table S1.

**Figure 4:**
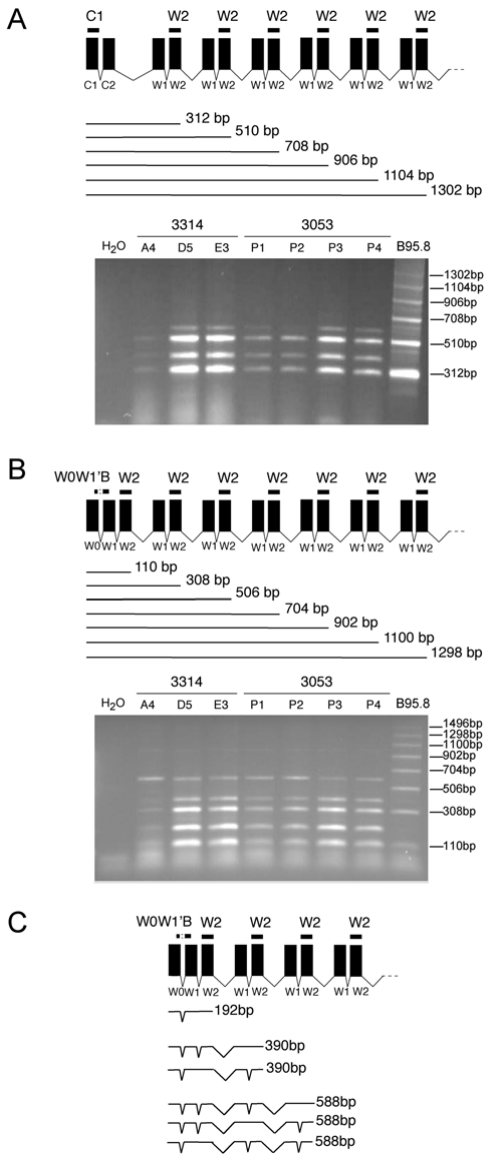
RT-PCR analysis of the Cp and Wp promoters indicated incorrect splicing and premature termination of viral EBNA transcripts in mESCs. RT-PCR analyses of EBNA-specific transcripts with primer pairs, which selectively amplify Cp or Wp originating transcripts. The expected patterns of multiply spliced exon combinations are schematically depicted. (A) C1/W2 primer pairs amplify transcripts, which originate from Cp. (B) W0W1'B/W2 primer pairs detect transcripts from Wp. The agarose gels show the RT-PCR patterns of different EBV-transduced Bruce4 EBNA1 clones together with the positive controls derived from B95.8 cells. (C) Off-size PCR products were isolated from gels as shown in B and directly sequenced. Their composition is schematically shown.

*EBNA2* and other members of the *EBNA* gene family (*EBNA-LP*, *EBNA3A*, *-B*, *-C*, and *EBNA1*) are expressed from multiply spliced transcripts, which originate from the Cp and/or Wp promoters (Fig. 2). By RT-PCR, we analyzed the activities of the Cp and Wp promoters with appropriate primer pairs (Fig. 4A, B and Table. S1). The positive control with cDNA derived from the B95.8 cell line gave the characteristic ladder of orderly spliced exon repeats (Fig. 4A, B). Cp- and Wp-specific cDNAs could be readily detected in EBV-transduced mESCs but the amplified cDNAs revealed only two of the expected PCR products (312 bp and 510 bp with Cp-specific primers; 110 bp and 308 bp with Wp-specific primers; Fig. 4A, B) in contrast to B95.8. Moreover, the PCR products indicative of longer, multiply spliced adducts were absent in EBV-positive mESCs while additional, smaller and prominent bands appeared which were not present in B95.8 cells (Fig. 4A, B). The unexpected bands were isolated from the gels and directly sequenced. The three aberrant bands amplified with the Wp-specific primer pair were 192, 390, and 588 bp in size and contained intron sequences as schematically shown in Fig. 4C; similar to the unexpected Cp-specific fragments (Fig. 4B and data not shown). It thus appears that alternative RNA splicing in mESCs resulted in retained introns in common EBNA transcripts. In addition, premature termination of aberrantly spliced mRNAs was evident. It is likely that both processes contributed to the failure of these cells to express *EBNA2* and other members of the *EBNA* gene family, including *EBNA1*.

### *In vitro* B cell differentiation of mECSs

**Figure 5:**
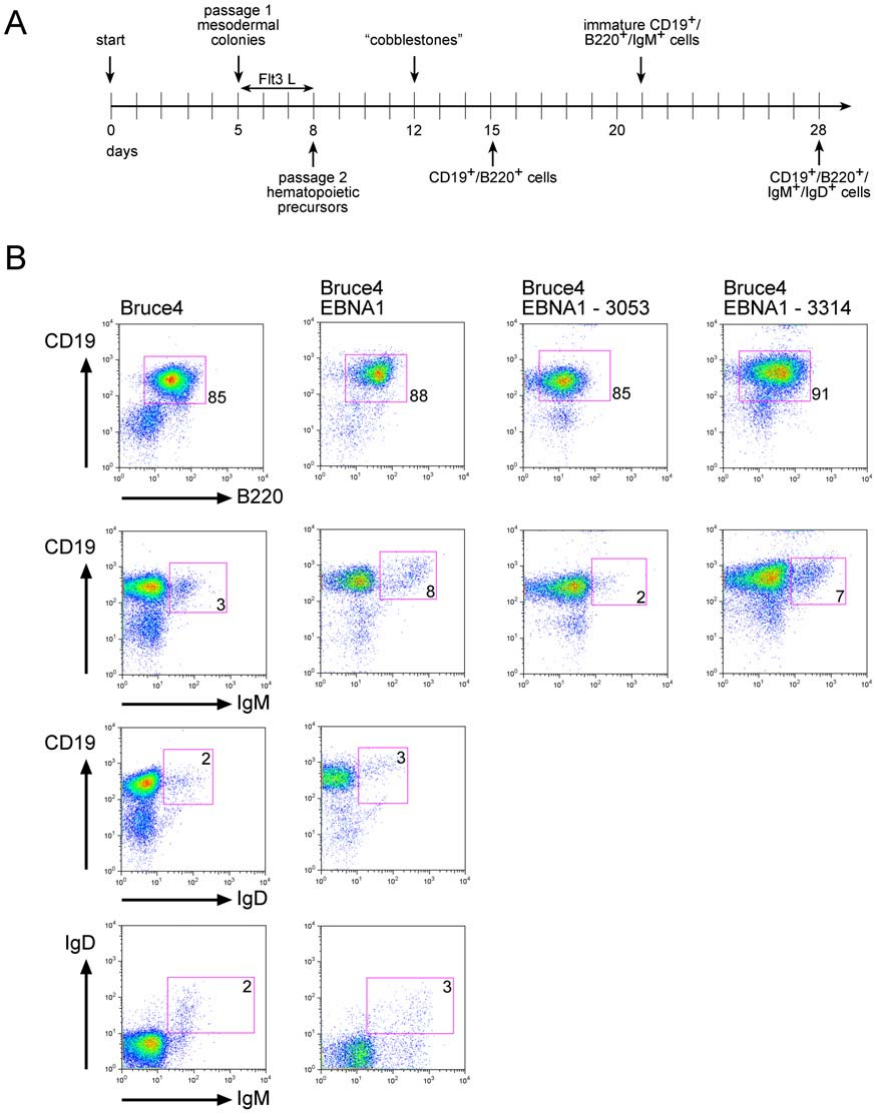
*In vitro* differentiation of EBV-transduced mESC lines. (A) Schematic protocol of the different steps, which lead to *in vitro* differentiated murine B cells. Cobblestone indicates the appearance of cell areas in which hematopoietic precursors develop. (B) Flow cytometry analysis of selected cell cultures gated on lymphocytes of EB5 mESCs, the Bruce4 EBNA1 clone C1 and two derivatives transduced with the recombinant EBV genomes p3053 and p3314 as indicated. Numbers indicate the percentage of B cells (B220^+^/CD19^+^), IgM^+^ B cells and IgM^+^/IgD^+^ B cells within the lymphocyte gate. Cells were analyzed between day 20 and day 23 of the differentiation protocol.

Our final goal was to study EBV's phenotype in mouse B cells *in vitro*. We also expected that viral genes like *EBNA2*, which were not expressed in mESCs (Fig. 3C), might be expressed upon differentiation to cells of the B-cell lineage. mESCs can be differentiated to mature B cells *in vitro* [20], [21]. We optimized the published protocols to improve the rate of B cell differentiation (Fig. 5A) to obtain sufficient numbers of B cells from the originally described EB5 mESC line, EBNA1-expressing Bruce4 mESCs and derivatives carrying the recombinant maxi- and mini-EBV genomes. Drug selection for the maintenance of the recombinant EBV genomes could not be applied during the differentiation process because OP9 feeder cells are sensitive to G418. The recombinant EBV genomes were stably maintained in mESCs for several weeks without selection (data not shown) and under conditions of mESC differentiation (below).

The EB5 mESC line, Bruce4 EBNA1 C1 cells and several derivatives carrying the recombinant EBV genomes p3053 or p3314 were cultivated according to the protocol shown in Fig. 5A with similar results. The efficiencies of B cell differentiation with EB5 and Bruce4 EBNA1 mESCs were comparable. Both immature (IgM^+^) and mature (IgM^+^/IgD^+^) B cells arose by about three weeks of differentiation. Representative examples are shown in Fig. 5B. Generally, in the lymphocyte gate >80% of viable cells were CD19^+^/B220^+^ B cells; the majority of this B cell population was IgM^−^ (pro/pre B cells) and minor populations of up to 8% and 3% expressed IgM and IgD respectively.

On day 21 of culture, the differentiated mESCs were separated into two subpopulations, IgM surface-positive and -negative cells by appropriate antibodies and selection with magnetic beads. From the IgM^+^ and IgM^−^ cell populations cellular DNA and RNA were isolated, cDNAs were prepared and expression of the three viral latent genes *EBNA2*, *LMP1* and *LMP2A* was analyzed by real-time PCR. The results are summarized in Table 2. EBV DNA was readily detectable in both populations, demonstrating that the mini- and maxi-EBV genomes were maintained during B cell differentiation for weeks. Parallel to our findings in mESCs, *LMP1* and *LMP2A* were clearly expressed in the IgM^+^ fraction and EBNA2 was undetectable. IgM^−^ cell populations consisted of 20–30% B cells (IgM^−^ pro/pre B cells; data not shown). In IgM^−^ cells, LMP2A- and EBNA2-specific transcripts were absent and LMP1-specific PCR products were inconsistently detectable (Table 2). The numbers of cells, which we could generate *in vitro* limited the amount of RNA preventing a detailed analysis of the activities of the Cp and Wp promoters.

**Table 2:** Results of RT-PCR analysis of *in vitro* differentiated murine B cells transduced with two recombinant EBV genomes.

	EBV DNA	GAPDH	EBNA2	LMP1	LMP2A
Bruce4 EBNA1 3314 A4 IgM^+^	+	+	–	+	+
Bruce4 EBNA1 3314 A4 IgM^−^	+	+	–	+	–
Bruce4 EBNA1 3053 P1 IgM^+^	+	+	–	+	+
Bruce4 EBNA1 3053 P1 IgM^−^	+	+	–	–	–
B95.8	+	+	+	+	+

(+) indicates detection of cDNA of a given transcript, minus (−) its absence. B95.8 cells serve as positive control for EBV's latent genes. The gene encoding glyceraldehyde 3-phosphate dehydrogenase (GAPDH), is an ubiquitously expressed housekeeping gene, which serve as another positive control for correct cDNA synthesis and PCR amplification. EBV (+) indicates detection of genomic EBV DNA. Primers used are listed in Table S1.

We interpret the lack of *EBNA2* expression to mean that similar to EBV-positive mESCs, abberant mRNA splicing and/or premature termination blocked the expression of this gene and presumably that of other members of the *EBNA* family. Remarkably, we never observed the outgrowth of murine B cells to yield lymphoblastoid cell lines; such lines readily originate from EBV-infected human B cells of any stage of differentiation including immature and even precursor B cells lacking rearranged immunoglobulin genes [2], [22]–[24]. The expression pattern of EBV's latent genes in mESCs or IgM^+^ B cells was reminiscent of the latency II program observed in human tumors and *in vitro* EBV-infected B cell lymphoma cell lines [25], [26]. In summary, EBV-transformed murine B cells did not arise in this *in vitro* model, presumably due to their failure to express the latent *EBNA* genes.

## Discussion

EBV has been known for more than 40 years but a small-animal model, which would allow the study of different aspects of EBV's biology and its contribution to malignancies is still lacking. A mouse susceptible to infection with EBV would constitute such a model, but murine cells are refractory to infection by EBV. Several attempts to overcome this obstacle by e.g. expressing human CD21 and/or human HLA class II molecules in primary murine B cells *in vitro* or in transgenic mice have met with limited success [13], [14] (R. Longnecker, personal communication). It is unclear why these approaches did not yield the desired mouse model but it is clear that properties in addition to the successful early steps of infection are required for such a model.

More is known about the maintenance (i.e. nuclear retention and DNA replication) of the EBV genome in murine cells. Upon transduction of recombinant EBV DNA, the viral genome is stably and extrachromosomally maintained in murine B cell lines engineered to co-express human CD21 and HLA class II [13] indicating that mouse B cells have the ability to support extrachromosomal EBV genomes similar to human cells [27]. EBNA1 is the indispensable viral factor, which mediates nuclear retention and replication of the EBV genome; but EBNA1 also transactivates other *EBNAs* [8], [28], [29]. We had found that mESCs could be easily transfected with genomic EBV DNA but their selection did not lead to stable cell lines retaining EBV. We suspected EBNA1 to be needed and engineered an *EBNA1*-positive mESC line (Fig. 1). Upon DNA transfection, all recombinant EBV genomes conferred G418 resistance (Fig. 3) and, once established, were even maintained for up to four weeks without selection (Table 2). All EBV-transfected EBNA1-mESCs were microscopically normal (Fig. 3A) and expressed *Nanog* and *Oct4* (Fig. 3C) indicative of their undifferentiated, pluripotent state [19]. Thus, introducing a fully transformation-competent EBV genome into embryonic stem cells does not reveal a profound phenotype.

It was evident that mESCs neither supported expression of *EBNA1* as indicated by our initial observations nor *EBNA2* as revealed by PCR analysis (Fig. 3C, Table 1). *EBNA1* can be expressed from three different promoters: Qp, Wp, or Cp; other members of the *EBNA* family (*EBNA3A*, -*3B*, -*3C*, *EBNA-LP*) are transcribed from Wp or Cp (Fig. 2). We did not analyze Qp's activity but Wp and Cp were clearly transcriptionally active (Fig. 4). The 5'-ends of viral EBNA transcripts, which originate from Cp or Wp consist of an array of small exons, which are multiply spliced. The typical ladder of PCR products reflects the sequential assembly of W1 and W2 exon pairs, which stem from each copy of the so-called BamHI-W-repeats (Fig. 2) as demonstrated in B95.8 cells (Fig. 4). Although p3053 and p3314 differ in the number of their BamHI-W-repeats, identical patterns of PCR products were detected (Fig. 4A, B). PCR products obtained with Wp-specific primer pairs (Fig. 4B) revealed only two out of five bands, which comigrated with those from B95.8 cells. These two bands were 110 and 308 bp in length as expected for transcripts with one and two BamHI-W-repeats, respectively (Fig. 4B), the remaining bands were composed of incompletely spliced products schematically depicted in Fig. 4C. No correctly spliced transcripts exceeding 400 bp could be detected (Fig. 4A, B) even in mESCs stably transduced with p3054, which carries about ten copies of the BamHI-W-repeats (Fig. 2). It thus appeared that intron retention within the 5′ end of EBNA transcripts and premature termination of Cp and Wp transcripts abrogated expression of *EBNAs* in mESCs. Clearly, intron retention can downregulate expression of the encoding gene [30] or inhibit its translation [31].

Alternative splicing is differently regulated in embryonic stem cells as compared to hematopoietic cells but not necessarily conserved between orthologous genes in mouse and man [32]. Therefore, we hoped that expression of *EBNA* genes might resume upon differentiation of mESCs to murine B cells. B cells presumably also support epigenetic changes of latent EBV genomes [33], similar to epigenetic modifications during differentiation of ESCs into somatic cells [34].

We improved the protocol for differentiation of mESCs into B cells, so that in some experiments even mature IgM^+^/IgD^+^ B cells could be detected (Fig. 5B). Because the differentiated B cells maintained the transduced EBV genomes (Table 2), we hoped that upon B-cell differentiation murine lymphoblastoid cell lines might arise, as is the case with human B cells infected with EBV. However, no such cell line could be obtained. The results summarized in Table 2 clearly indicated that IgM^+^ cells expressed only *LMP1* and *LMP2A* but not *EBNA2* similar to *in vitro* infected murine B cell lymphoma cell lines [13]. We concluded that this unexpected failure prevented the outgrowth of EBV-transformed lymphoblastoid cell lines. Unfortunately, we did not obtain sufficient RNA for a more comprehensive analysis of transcripts originating from Cp or Wp but, given our findings with mESCs, we hypothesize that aberrant RNA splicing and/or premature termination of transcripts was responsible for this outcome. It would be interesting to know whether such splicing alterations arise as a result of altered transcription due to epigenetic marking of the viral genome in murine cells, e.g. DNA methylation or heterochromatin formation in and around the BamHI-W-repeat region.

To our knowledge no report describes *in vitro* differentiation of human embryonic stem cells (hESCs) to B cells, thus a positive control in parallel to our experiments with mESCs was not feasible. EBV-derived plasmids can be maintained in hESCs [35] and according to published reports, EBV is capable of transforming human B cells of all stages of differentiation including progenitor, pro- and pre-B cells [22]–[24]. These reports suggest that our experimental approach could theoretically be extended to hESCs to give rise to classical lymphoblastoid cell lines. One of our initial goals was to study EBV in recipient B cells with different genetic backgrounds. Because hESCs can be genetically altered, our strategy could be adopted to hESCs assuming that they will give rise to any cell of the B cell lineage.

In conclusion, fundamental differences in gene regulation and presumably processing of mRNAs between mouse and man appears to block the route towards a tractable murine model for EBV. It is not obvious to us how this unexpected problem could be solved except by ectopic expression of EBV's *EBNA* genes in mouse B cells.

## Materials and Methods

### Cell lines and culture condition

Bruce4 mouse embryonic stem cells (mESC) are derived from the C57/BL6 mouse strain and were cultured in DMEM and 10%FCS with 1.2 mM sodium pyruvate, 1.2 mM glutamine and 0.12 mM β-mercaptoethanol complemented with 1.2x non-essential amino acids and 1800 u/ml ESGRO (Chemicon). Murine embryonic fibroblasts (mEF) isolated from transgenic mouse embryos carrying the gene for neomycin phospho-transferase (*neo*) were used as feeder cells and seeded on dishes covered with 2% gelatine in PBS. Prior to plating of mESCs, mEF were mitotically inactivated with mitomycin C (10 µg/ml) for 2–4 h. OP9, a stromal cell line derived from newborn op/op mice carrying an inactivating mutation in the *M-CFS* gene [*20*] was cultured in α-MEM, 20%FCS, 0.1 mM β-mercaptoethanol. B95.8, a lymphoblastoid monkey cell line, which releases the prototypic EBV B95.8 virus [*36*] and Raji, an EBV-positive Burkitt Lymphoma cell line [*37*], were maintained in RPMI1640 medium with 10%FCS, 100 µg/ml streptomycin, 100units/ml penicillin G, 1.2 mM sodium pyruvate, and 10 mM HEPES.

### Primers used in quantitative real-time PCR (qRT) and RT-PCR

PCR detection of viral DNA was performed with the oligonucleotide primer pair 2190 B2 and 2190 F3. cDNAs specific for viral transcripts encoding the *EBNA2, LMP1, LMP2A* genes, and the complex patterns of transcripts originating from the Cp and Wp promoters, which drive expression of the different *EBNA* genes, were assessed with appropriate primer pairs listed in Table S1. PCR detection of mouse GAPDH or actin cDNAs served as a positive control.

### DNA extraction, restriction enzyme cleavage, and Southern blot analysis

Isolation of cellular DNA, and Southern blot hybridisations were performed according to standard protocols. DNA probes were labeled with [α^32^P] dCTP with High Prime probe staining kit (Roche). After probe denaturation (95°C for 5 min), the membranes were hybridized in Church buffer at 65°C overnight. The blots were washed in 0.2xSSC/1%SDS buffer at 60–65°C. Autoradiography was carried out at –80°C with intensifying screens (Biomax MS).

### Protein isolation and Western blot analysis

Cells were lysed in Laemmli buffer (2.5% SDS, 20 % glycerol, 0.12 M Tris pH 6.8) supplemented with protease inhibitor Complete Mini Tablets (Roche). Protein concentrations were determined with the DC Protein Assay (Bio-Rad). Protein samples were electrophoretically separated on SDS-PAGE gels, transferred to Hybond-C membranes (Amersham Pharmacia) and incubated with a primary antibody for EBNA1 (1H4) and a secondary horse radish-conjugated anti-rat goat antibody (Dianova). Chemiluminescent signals were detected with ECL reagent (Amersham Pharmacia).

### Plasmid DNA isolation, DNA transfection, and an establishment of mESC lines

Plasmids up to 30 kb were propagated in the DH5α E.coli strain and extracted with the Jet Star DNA Isolation Kit (Genomed). Large EBV plasmids were propagated in DH10B and isolated with a modified alkaline lysis protocol. Plasmid DNA was purified on cesium chloride/ethidium bromide gradients, precipitated with isopropanol and dissolved in TE. 1×10^7^ mESCs were electroporated (2.3 kV, 500 µF) in 700 µl RPMI 1640 with 25 µg plasmid DNA. After 48 hours, the cells were selected with G418 (0.4 mg/ml) for up to 11 days. Single colonies were picked in PBS and trypsinized prior to plating on 96-well plates.

### Gardella gel assays

5×10^6^ cells in 50 µl of 15% Ficoll were loaded per slot on a 0.8% agarose gel in TBE (89 mM Tris-HCl, 89 mM Borate, 2 mM EDTA). The gel slice above the slots consisted of agarose containing 2% SDS and 1 mg/ml proteinase K to allow lysis of the cells and release of intact extrachromosomal DNA. Gels were run at 4°C at 20 V for 3 h and overnight at 120 V. DNAs were detected by Southern blot analysis.

### RNA isolation, reverse-transcription and PCR analysis

RNA was isolated with the RNeasy Midi Kit (Qiagen). 2–5 µg of total cellular RNA was reverse-transcribed with the cDNA Synthesis System (Invitrogen). Oligo-(dT)_20_ primers were denatured for 5 min at 65°C and mixed with 10x RT-buffer (25 mM MgCl_2_, 0.1 M DTT, 40units/µl RNase) and Superscript™III-Reverse Transcriptase. The reaction was carried at 50°C for 50 min and terminated by heating at 85°C for 5 min. Samples were incubated with 2units/µl RNaseH at 37°C for 20 min. For real-time PCR, cDNA obtained from 2.5–5 µg RNA was diluted 1∶10 and analyzed in the LightCycler machine (Roche). 10 µl of the reaction consisted of 1 µl cDNA, 0.8 µl MgCl_2_ (25 mM), 50 pmol of each primer, 1 µl polymerase mix and water. The PCR reactions were carried out for up to 55 cycles. For RT-PCR, the following conditions were applied. Each sample contained 2 µl cDNA, 1x KCl buffer, 0.2 mM dNTPs mix, 50 pmol of each primer, 3 units Taq-DNA-Polymerase (Fermentas), 0.2 µl DMSO and 1–1.5 mM MgCl_2_ for up to 35 cycles.

### *In vitro* B cell differentiation

The published differentiation protocol [**21**] was modified as follows: On day 0, 1×10^4^ mESCs were plated in one well of a six-well cluster plate in differentiation medium (α-MEM, 10% FCS, 0.1 mM β-mercaptoethanol) on OP9 feeder cells. The medium was exchanged on day 3. On day 5 loosely adherent cells were transferred to a new well with OP9 feeder cells to remove undifferentiated mESCs and highly proliferative mesenchymal cells. Medium supplemented with Flt3L (20 ng/ml) was added for three additional days. Non-adherent small cells were transferred to two wells of a six-well cluster plate and cultivated on OP9 feeder cells until up to day 21 but in the absence of Flt3L. When B220^+^/CD19^+^/IgM^+^ cells were identified in the supernatant, the cell culture was kept until day 28 of the experiment to allow B cell maturation to IgD^+^ cells.

### FACS analysis and MACS cell sorting

Prior to cytometry cell samples were washed with PBS/2%FCS and incubated with suitable antibodies conjugated to fluorescent dyes (anti-B220-RA3-6B2, 1∶200, mouse, PerCp; anti-B220-RA3-6B2, 1∶250, mouse, APC; anti-CD19-1D3, 1∶400, mouse, PE; anti-IgM-II/41, 1∶200, mouse, APC; anti-IgD-11-26c.2a, 1∶800, mouse, FITC; all by Pharmingen) diluted in PBS/2%FCS. Dead cells were stained with propidium iodide. When possible, 5×10^4^ lymphocytes (defined by the lymphocyte gate) were analyzed per sample. IgM^+^ cells were separated by magnetic cell sorting (Miltenyi Biotec) on day 21. All cells in the flow-through were considered as IgM^−^ population.

## Supporting Information

Table S1 (0.04 MB DOC)
