# Supplementary material for: Restricted Expression of Epstein-Barr Virus Latent Genes in Murine B Cells Derived from Embryonic Stem Cells
Source: PLoS One. 2008 Apr 16;3(4):e1996. doi: 10.1371/journal.pone.0001996 (PMC2289878; doi:10.1371/journal.pone.0001996)
Supplement: Table S1 — (0.04 MB DOC) [file pone.0001996.s001.doc]

Table S1. List of primers used in real-time PCR and RT-PCR.

| Primer name | Sequence 5’ to 3’ |
| --- | --- |
| 2190 B2 | CCCGTCATTCCCGTCGTG |
| 2190 F3 | ATCTGCTTCTGGCTCTTCTGGG |
| 89 mGAPDH seq | TCGTCCCGTAGACAAAATG |
| 90 mGAPDH rev | GAGATGATGACCCGTTTGG |
| EBNA2-RTas | TGTCTGACAGTTTTCCTGGTAGG |
| EBNA2-RTse | AGAGTGGCTGCTACGCATTAGAG |
| LMP1-RTas | TGTTCATCACTGTGTCGTTGTCC |
| LMP1-RTse | AGAAGAGACCTTCTCTGTCCACT |
| LMP2A-RTas | CATGTTAGGCAAATTGCAAA |
| LMP2A-RTse | ATGACTCATCTCAACACATA |
| C1 | TGTAGATCTGATGGCATAGAGAC |
| W2 | ACTGAAGCTTGACCGGTGCCTTCTTAGGAG |
| W0W1´B | GGAGTCCACACAAATGGG |
